# Urban-rural disparities in health care utilization among Chinese adults from 1993 to 2011

**DOI:** 10.1186/s12913-018-2905-4

**Published:** 2018-02-09

**Authors:** Jiajia Li, Leiyu Shi, Hailun Liang, Gan Ding, Lingzhong Xu

**Affiliations:** 1 School of Public Health Shandong University, Jinan, People’s Republic of China; 2 Johns Hopkins Bloomberg School of Public Health, Baltimore, USA

**Keywords:** Urban/rural, Hukou system, Health care seeking behaviour, Dynamic trends, China

## Abstract

**Background:**

Despite economic growth and improved health outcomes over the past few decades, China still experiences striking urban-rural health inequalities. Urban and rural residents distinguished by the hukou system may experience profound disparities because of institutional effect. The aim of this study is to estimate trends in urban-rural disparities in self-care, outpatient care, and inpatient care utilization from a perspective of the hukou system.

**Methods:**

Data were extracted from the seven latest waves of the China Health and Nutrition Survey (CHNS). We used the hukou system to distinguish between urban and rural residents. Chi-square tests were performed to examine urban-rural gaps in self-care, outpatient care, and inpatient care utilization. Multinomial logistic regression was employed to confirm these disparities and to explore whether the urban-rural gaps have narrowed or widened from 1993 to 2011 once known determinants of utilization are taken into account according to Andersen/Aday’s Health Behaviour Model.

**Results:**

The urban-rural disparities were evident after controlling for confounding variables: urban adults were 3.24 (*p* < 0.05), 2.23 (*p* < 0.1), and 4.77 (*p* < 0.01) times more likely to choose self-care vs. no care, outpatient care vs. no care, and inpatient care vs. no care than their rural counterparts, respectively. The results showed upward trends in self-care, outpatient care, and inpatient care utilization from 2004 to 2011. The urban-rural gaps in health care utilization gradually narrowed during the period of 1993–2011. The hukou distinctions of self-care, outpatient care, and inpatient care in 2011 were only 33.3%, 35.5%, and 9.6% of that in 1993, respectively.

**Conclusions:**

Although rural residents were underutilizing health care when compared to their urban counterparts, the significant decrements in urban-rural disparities reflect the positive effect of the on-going health system reform in China. To maintain an equitable distribution of health care utilization, policy makers need to be aware of challenges due to aging problems and health expenditure increment.

## Background

Ensuring equitable access to health services is an enduring concern for health system planners and policy-makers [1]. Despite economic growth and improved health outcomes over the past few decades in China, previous studies indicate significant urban-rural inequality in health related issues such as health care resources [2, 3], health outcomes in adults and children [3–6], and prevalence of certain diseases [7–12]. Compared with urban residents, infant mortality in the rural population was over 2 times higher (5.2 of 1000 vs. 12.4 of 1000) and health expenditures per capita were nearly one third lower (2969 yuan vs. 1056 yuan) in 2012 [13]. Although many factors account for the large gap between urban and rural environments, access to the health care system is a pivotal factor [14]. Equitable utilisation of health care is now a research priority in China [1].

A number of studies have compared the differences between rural and urban residents in the utilization of medical care services. Research has generally found lower utilization of all sorts of health care services in the rural area [1, 15–22], due to the unbalanced development between urban and rural areas in medical service supply. Urban–rural health disparities are expected to be even more pronounced in China since the Chinese government enforces a household registration system (hukou) to control internal migration. The hukou system, established in 1955, classified all residents into agricultural (rural) and non-agricultural (urban) registration categories. During the era of planned economy (1956~ 1978), the policy favoured urban hukou holders in many ways such as employment opportunities, ration stamps, housing, and subsidized services. Following China’s economic transition and urbanization in the 1980s, a large number of rural residents moved to cities without converting their hukou status. During period of this study (1993~ 2011), although the government gradually eased the hukou regulations, rural residents still encountered barriers in obtaining basic public services and welfare such as healthcare, education system, and social security coverage [23, 24]. For example, the social health insurance system has been separated into the urban system and rural system by hukou. Urban employees and unemployed residents respectively obtain health insurance through programs called Urban Employee Basic Medical Insurance (UEBMI) and Urban Resident Basic Medical Insurance (URBMI), whereas rural hukou holders are stipulated to participate in a community health insurance scheme known as the New Cooperative Medical System (NCMS) [25, 26]. Compared with the urban insurance, rural health insurance has inferior health coverage and inconvenient reimbursement procedures, which result in potential barriers for rural residents to obtain appropriate and cost-effective health care [27, 28]. Therefore, we estimate urban-rural disparities from the perspective of hukou in this study.

Health care utilization is the most activity-related and consumer-oriented problem in planning health care delivery [21]. The Chinese government conducted an array of reforms aimed at narrowing urban-rural disparities since 1997 [9]. Previous studies, which have been based on a cross-sectional or early period database [29–32], may not reflect the longitudinal effects of the reform activities. Given this background, our study seeks to meet three objectives: first, to explore the extent to which rural and urban patients differed in health care utilizing behaviour in view of hukou; second, to determine how much of the variation in health care utilization remains once other determinants are accounted for; and third, to examine trends of urban-rural disparities in health care utilization by capitalizing on a longitudinal database from 1993 to 2011.

## Methods

### Data

Seven waves of data from the China Health and Nutrition Survey (CHNS) were used to study urban–rural disparities in health care utilization among Chinese Adults from 1993 to 2011. The CHNS is an ongoing project held by University of North Carolina Chapel Hill and the Chinese Centre for Disease Control and Prevention, dating back to1989. Although nine waves of data have been publicly available, the hukou information has been only collected in waves beginning in 1993. Thus, we used CHNS 1993–2011 in this study and focused on adults above the age of 18 who had been sick, or suffered from a chronic or acute disease during the previous 4 weeks.

### Framework

We assumed that individuals who reported being sick during the last 4 weeks were faced with four different alternatives including: not seeking care, self-care, outpatient visit, and inpatient visit. Thus, health care utilization was measured as self-care utilization, outpatient utilization, and inpatient utilization, where no care was used as a reference group in the estimation. In particular, self-care (such as taking medication stored at home or purchased at a pharmacy) was considered as one of the major ways to use health care, because a prescription was not required when purchasing drugs at a pharmacy before 2006 in China [29]. Each eligible pharmacy is staffed with 1–2 pharmacists or visiting doctors, who can provide simple consultation and referral services for patients. The drug cost in certificated pharmacies could also be partly covered by individual saving accounts (ISA) of health insurance [33].

The utilization of health services could be viewed as a type of individual behaviour [34]. We used the Andersen/Aday Health Behaviour Model to assess whether hukou bears relation to individuals’ health care utilization [35–38]. According to the model, usage of health services is determined by need factors, enabling factors, and predisposing factors. The association we were trying to test can be expressed in the following general equation:

1 $$ {\mathrm{hcu}}_{\mathrm{i}}=\mathrm{f}\left({\mathrm{hn}}_{\mathrm{i}},{\mathrm{he}}_{\mathrm{i}},{\mathrm{pd}}_{\mathrm{i}},{\mathrm{HUKOU}}_{\mathrm{i}},{\mathrm{wave}}_{\mathrm{i}},{\mathrm{HUKOU}}_{\mathrm{i}}\ast {\mathrm{wave}}_{\mathrm{i}}\right) $$

Where *hn*_*i*_ was a vector of health need characteristics, including categorical variables for illness/injury severity (not severe, somewhat severe, quite severe), a dummy variable indicating whether individuals had been diagnosed having diseases including hypertension, diabetes, myocardial infarction, stroke or transient ischemic attack, cancer, bone fracture, asthma (disease history); *he*_*i*_ was a vector of health enabling characteristics, including education (formal education years), income proxy by per capita per year household income in 2011 value, categorical dummy variables for health insurance (NCMS, URBMI, UEBMI; others including commercial medical insurance, government free medical insurance), categorical variables for area (western, central, north-eastern, eastern); *pd*_*i*_ was a vector of predisposing characteristics, including a continuous age variable (age), a dummy variable for gender (female), and a binary variable for marital status (married). In particular, as the key explanatory variable, the dichotomous variable HUKOU was singled out, assessed with a question: “To which type of household registration do you belong, urban or rural?” To capture how health care utilization and urban-rural disparities shifted over time, we included categorical variables *wave*_*i*_ indicating time periods (1993, 1997, 2000, 2004, 2006, 2009, and 2011) and an interaction term *HUKOU*_*i*_ ∗ *wave*_*i*_into model (1).

### Statistical analyses

We began our analysis by comparing measures of health care utilization as well as other explanatory variables between urban and rural samples, using pooled cross-sectional CHNS data for 1993–2011. χ^2^ Tests for dichotomous variables and t-tests for continuous variables were used to assess whether the urban–rural differences were statistically significant; we also reported their *p*-values. Due to the nature of the dependent variable, multinomial logistic (MNL) estimation was employed to study urban-rural disparities of health care utilization after controlling for the confounding variables, and to check their statistical significance level. Relative risk ratios (RRR) and their *p*-values were also reported. We also test the independence of irrelevant alternatives (IIA) by using Hausman-McFadden test [39] to avoid inconsistent parameter estimates due to non-compliance with IIA.

## Results

### Descriptive results

According to Table 1, statistically significant differences between rural and urban adults were observed for all the characteristics. Table 1 demonstrated that on average rural adults were slightly younger, with a larger proportion of female to male, and a lower level of education and income. Rural individuals also tended to be married, had less disease history, and large proportion were severely sick. The summary statistic also illustrated that individuals who lived in western and central areas were more inclined to be rural hukou holders, whereas more urban individuals lived in north-eastern and eastern regions. The main difference between urban and rural subjects was that most of the rural subjects joined in NCMS whereas most of the urban subjects participated in UEBMI and URBMI. Furthermore, rural individuals had a larger proportion of uninsured and smaller proportion of other insurances.

**Table 1:** Descriptive statistics

Variable	All	Rural	Urban	*P*
What did you do when sick?, n (%)				0.000
Did not pay any attention	957(9.49)	583(10.75)	374(8.03)	
Self-care	2650(26.29)	1141(21.04)	1509(32.40)	
Outpatient	5860(58.14)	3398(62.67)	2462(52.87)	
Inpatient	612(6.07)	300(5.53)	312(6.70)	
Health need variables				
How severe was the illness or injury?, n (%)				0.000
Not severe (Ref.)	4118(40.86)	2256(41.61)	1862(39.98)	
Somewhat severe	4848(48.10)	2507(46.24)	2341(50.27)	
Quite severe	1113(11.04)	659(12.15)	454(9.75)	
Diseases history, n (%)				0.000
No (Ref.)	6547(64.96)	3936(72.59)	2611(56.07)	
Yes	3532(35.04)	1486(27.41)	2046(43.93)	
Predisposing variables				
Age, mean (SD)	55.61(15.45)	54.35(15.20)	57.07(15.62)	0.000
Gender, n (%)				0.001
Male (Ref.)	4313(42.79)	2236(41.24)	2077(44.60)	
Female	5766(57.21)	3186(58.76)	2580(55.40)	
Marital status, n (%)				0.001
Married (Ref.)	8116(80.52)	4433(81.76)	3683(79.09)	
Others	1963(19.48)	989(18.24)	974(20.91)	
Health enabling variables				
Education, mean (SD)	6.44(4.62)	4.99(3.93)	8.14(4.79)	0.000
Income (RMB in 2011 value), mean (SD)	10,221.41 (12,190.99)	7098.18 (9421.41)	13,857.69 (13,919.75)	0.000
Types of medical insurance, n (%)				0.000
None (Ref.)	3810(37.80)	2360(43.53)	1450(31.14)	
NCMS	3086(30.62)	2791(51.48)	295(6.33)	
URBMI	744(7.38)	55(1.01)	689(14.79)	
UEBMI	1156(11.47)	50(0.92)	1106(23.75)	
Others	1283(12.73)	166(3.06)	1117(23.99)	
Area (%)				0.000
Western (Ref.)	2832(28.10)	1832(33.79)	1000(21.47)	
Central	3009(29.85)	1776(32.76)	1233(26.48)	
North-eastern	1503(14.91)	792(14.61)	711(15.27)	
Eastern	2735(27.14)	1022(18.85)	1713(36.78)	
Wave, n (%)				0.000
1993 (Ref.)	343(3.40)	204(3.76)	139(2.98)	
1997	656(6.51)	368(6.79)	288(6.18)	
2000	695(6.90)	367(6.77)	328(7.04)	
2004	1854(18.39)	1085(20.01)	769(16.51)	
2006	1551(15.39)	968(17.85)	583(12.52)	
2009	2094(20.78)	1190(21.95)	904(19.41)	
2011	2886(28.63)	1240(22.87)	1646(35.34)	

①χ^2^Tests for dichotomous variables and t-tests for continuous variables

It is worth highlighting the distribution of what individuals did when they got sick. Table 1 revealed that the majority of the patients chose outpatient care as their treatment option, with a larger proportion of rural to urban (62.67% vs. 52.87%, *P* < 0.001). Apart from outpatient, self-care was the most likely choice for sick individuals, with a larger proportion of urban to rural (32.40% vs. 21.04%). Rural residents also tended to choose no care and less likely to choose inpatient care when they got sick compared to their urban counterparts. Approximately 11% of rural adults did not pay attention to their sickness compared with 8% of the urban adults.

**Fig. 1:**
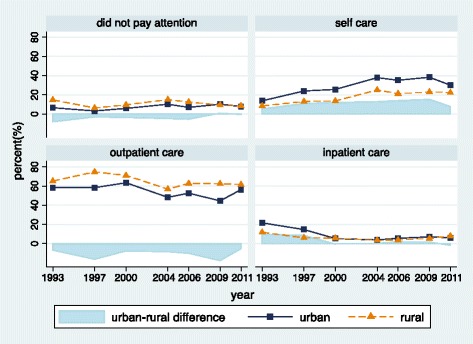
Healthcare utilization among urban and rural adults in China (1993–2011)

Figure 1 showed urban and rural trends in health care utilization from 1993 to 2011. Both rural and urban adults’ utilization in Fig. 1 showed a progressive increase in outpatient care from 1993 to 1997, after which there was a slight decrease until 2004. A gradual increase in self-care could be observed in both rural and urban groups, which was in contrast to that of inpatient care.

The greatest difference between urban and rural groups was found in self-care utilization. The raw urban-rural gap in self-care and outpatient care persisted during this period, which indicated different treatment preferences when the disease was not very severe.

Apparently, rural adults became progressively more likely to pay attention when they got sick after 2004, such as being more likely to choose inpatient care. The urban-rural disparities in no care and inpatient care also declined since the year 2004.

### Multivariate regression analyses

Table 2 reported the results of multinomial logistic regression for urban-rural disparities in health care utilization. No care was chosen to be the reference category. The Hausman test results in Table 3 showed that none of the four options would reject the IIA assumption.

**Table 2:** Multinomial logistic regression of Chinese adults’ health care utilisation using pooled CHNS data 1993–2011

	Self-care	Outpatient	Inpatient
	RRR	RRR	RRR
HUKOU: Urban	3.240**	2.234*	4.768***
	(1.671)	(0.942)	(2.557)
Wave: 1997	3.720***	3.193***	1.494
	(1.473)	(0.982)	(0.679)
Wave: 2000	2.574**	2.053***	1.045
	(0.962)	(0.572)	(0.451)
Wave: 2004	2.772***	0.913	0.275***
	(0.891)	(0.211)	(0.103)
Wave: 2006	2.826***	1.142	0.339***
	(0.934)	(0.276)	(0.133)
Wave: 2009	3.822***	1.390	0.583
	(1.318)	(0.359)	(0.240)
Wave: 2011	4.102***	1.394	0.896
	(1.428)	(0.365)	(0.367)
Urban*1997	0.826	0.540	0.545
	(0.553)	(0.315)	(0.394)
Urban*2000	0.789	0.553	0.206**
	(0.479)	(0.282)	(0.142)
Urban*2004	0.589	0.489^#^	0.270**
	(0.314)	(0.216)	(0.162)
Urban*2006	0.846	0.666	0.507
	(0.468)	(0.308)	(0.315)
Urban*2009	0.347*	0.262***	0.145***
	(0.189)	(0.119)	(0.087)
Urban*2011	0.333**	0.355**	0.096***
	(0.183)	(0.162)	(0.058)
Health need factors			
How severe was the illness or injury: Somewhat severe	1.417***	2.351***	5.715***
	(0.115)	(0.177)	(0.837)
How severe was the illness or injury: Quite severe	1.920***	4.411***	46.940***
	(0.349)	(0.746)	(9.877)
Diseases history: Yes	1.213**	1.371***	1.636***
	(0.113)	(0.119)	(0.206)
Predisposing factors			
Age	0.995^#^	0.995^#^	1.002
	(0.003)	(0.003)	(0.005)
Gender: male	0.974	1.032	0.825*
	(0.080)	(0.079)	(0.095)
Marital status: married	0.831*	0.727***	0.800^#^
	(0.079)	(0.064)	(0.113)
Health enabling factors			
Education	1.030**	1.010	1.002
	(0.012)	(0.011)	(0.016)
Household income per capita	1.000	1.000	1.000
	(0.000)	(0.000)	(0.000)
Types of medical insurance: NCMS	1.090	1.254*	1.462*
	(0.144)	(0.150)	(0.312)
Types of medical insurance: URBMI	1.301	1.579**	2.556***
	(0.277)	(0.313)	(0.793)
Types of medical insurance: UEBMI	1.652**	1.297	3.432***
	(0.324)	(0.239)	(0.993)
Types of medical insurance: others	1.384**	1.275*	2.241***
	(0.216)	(0.187)	(0.486)
Province: Central	0.762***	1.013	1.185
	(0.078)	(0.096)	(0.171)
Province: North-eastern	1.158	0.569***	0.690**
	(0.136)	(0.064)	(0.127)
Province: Eastern	0.707***	1.112	0.877
	(0.0815)	(0.117)	(0.144)
Constant	0.611	3.056***	0.129***
	(0.216)	(0.840)	(0.0558)
Observations	10,079	10,079	10,079
R-squared	0.081		

Robust standard errors are reported in parenthesis; *** *p* < 0.01, ** *p* < 0.05, * *p* < 0.1, # *p* < 0.15

**Table 3:** Hausman test results of the IIA

Omitted	chi2	df	*P* > chi2	evidence
0	− 4500.000	28	1.000	for Ho
1	− 4000.000	54	1.000	for Ho
2	−51.937	56	1.000	for Ho
3	− 510.629	28	1.000	for Ho

The urban-rural disparity was particularly evident in inpatient care utilization, after controlling for the confounding variables in eq. (1). The likelihood of choosing inpatient care vs. no care was 4.77 times greater for urban adults relative to rural adults when they got ill, which was statistically significant at the 1% level. Also, urban adults were 3.24 times more likely than their rural counterparts to choose self-care vs. no care (RRR = 3.240; *p* < 0.05) and were 2.23 times more likely to choose outpatient care vs. no care (RRR = 2.234; *p* < 0.1) when the confounding variables were held constant.

Results of health care utilization trends suggested a significant upward trend of self-care utilization, though there was a slight decrease in the 1997–2000 period. Compared to no care, after a sharp upward trend from 1993 to 1997, patients were less likely to use outpatient care from 1997 to 2004, and more likely to use outpatient care from 2004 to 2011. After the above-mentioned changes in trend, the probability of outpatient care vs. no care in 2011 was 1.394 times that of 1993. Similar trends could be discovered in inpatient care utilization. More specifically, the probability of inpatient care vs. no care in 2011 was even less than that of 1993.

Changes in urban-rural disparities could be clarified by the interaction between hukou dummy and wave dummies in Table 2. Decline trends of urban-rural disparities could be observed in self-care, outpatient care, and inpatient care from 1993 to 2011, though there was an increase from 2004 to 2006. More specifically, the downward trend of urban-rural disparities was the most obvious in inpatient care utilization, which was significant in most of the survey years. The hukou distinctions in self-care, outpatient care, and inpatient care in 2011 were only 33.3%, 35.5%, and 9.6% that of 1993, respectively.

Consistent with these findings, visible health care utilization disparities could be observed among different insurance types. The UEBMI participants were most likely to use self-care and inpatient care, while the URBMI participants were most likely to use outpatient care. In sum, the urban insurance enrolees had a higher likelihood of using health care than rural insurance enrolees.

Moreover, the severity of illness or injury and disease history had positive effects on all types of health care utilization, while age and marital status had negative effect on health care utilization. Adults with better education had a higher probability to choose self-care rather than no care.

## Discussion

The present study showed that more than half of patients chose outpatient care as their treatment option when they felt sick during the 4 weeks before the survey. However, although an insignificant increase in outpatient care utilization could be observed from 2004 to 2011 when holding everything else constant, the probability of choosing outpatient care was much lower than that before 2000. Instead, patients were more likely to use self-care since 2000. The most straightforward explanation for this phenomenon would be the sharp rise in health service costs. Between 1995 and 2011, average per capita health expenditures increased from 110.1 to 969.0 yuan among urban residents, and from 42.5 to 436.8 yuan among rural residents [13]. Some urban and many rural residents who could not afford such medical expenditures had to buy drugs from pharmacies [40], even for serious diseases like HIV/AIDS [41]. Additionally, the Chinese government has implemented the drug sales network in recent years, which highly improved the accessibility of pharmacies and medicines [40]. Some studies found that the aging population might be responsible for the increasing self-care utilization because seniors relied more on self-care to save the economic and time cost involved in seeking professional health care [42]. In developing countries, where heath service resources are scarce, despite being associated with risks [43], self-care should be an important supplemental approach to professional medical facilities. As the role of pharmacies in health care has been recognized by many countries [43–45], those pharmacies in China should take more responsibility for self-care monitoring and management.

We also concluded that rural hukou holders used less health care than their urban counterparts. Moreover, the hukou distinction in inpatient care utilization was more obvious than the distinctions in self-care and inpatient care utilization. These findings were consistent with earlier studies about urban-rural gaps in health care utilization [9, 29, 46, 47]. More specifically, rural residents had a higher proportion of outpatient care than urban residents in the descriptive results but had a lower probability of using outpatient care in the multivariate results after controlling for the confounding variables. The opposite results demonstrated that the urban-rural disparities on health care utilization were even more obvious than observed.

There are several potential explanations for the urban-rural disparities observed in this study. First, consistent with most previous studies from other countries, people in rural areas usually have a shortage of health care providers, extended travel, low socioeconomic status, and lack social support [1, 3, 48–51] [16]. Registered doctors per thousand populations in urban communities were 2.57 times more than in rural communities in China [13]. The second explanation is the different welfare system between urban and rural, due hukou. The major welfare benefit associated hukou is the social benefits system including health insurance [23, 52, 53]. The rural hukou holders are stipulated to participate in the local NCMS, which has lower benefit than that of the UEBMI and URBMI in urban areas. In 2008, the actual co-payment rates of UEBMI, URBMI, and NCMS were 36.8%, 50.7%, and 73.4%, respectively [54]. Under the two-class health insurance system, rural residents usually encounter more financial barriers in health care utilization. The average hospitalization expenses of the NCMS enrolees account for 56% of per capita annual income in 2008, while the proportions for UEBMI and URBMI enrolees are only 31.8% and 38.2%, respectively [54]. Such heavy burden compels rural patients to stay away from inpatient care, which can also explain why we could observe a larger urban-rural gap in inpatient care use than self-care and outpatient care use. Our findings suggested that extending health insurance coverage may have limited effect on promoting urban-rural equity, and integrating the decentralized insurance system into one uniform system should be taken into consideration. Moreover, the lack of inpatient health services provision in rural areas and the consequent long distance to gain access to appropriate treatment may also function as significant obstacles for rural residents to access and use inpatient health care.

An encouraging finding was that the urban-rural disparities in health care utilization had narrowed from 1993 to 2011, though not significantly before 2009. The continuous coverage expansion and benefit improvement of rural health insurance might have played a positive role in the gap narrowing. Between 1993 to 2013, the rural insurance coverage extended from 15.9% to 97.3% [54]. Besides, The central and local government subsidy increased from minimum 20 yuan in 2003 to 80 yuan in 2008, and to minimum 120 yuan in 2010 per enrolee per year [54]. Recently, immediate reimbursement from NCMS has been realized in more and more rural areas, which means NCMS enrolees only need to pay out-of-pocket for part of their medical expenditure [55]. All these reasons might work together to lower the financial barrier and to promote equal use of health care. More specifically, obvious decrements could be observed in self-care, outpatient care, and inpatient care since 2009, which might be due to the new nationwide health care system reform in China. In 2009, the central government of China started new health care reforms, which targeted universal and equitable access to health care by 2020 [46, 56]. Between 2009 and 2011, 850 billion yuan had been spent in expanding health insurance coverage, improving primary care, establishing essential medicines system, promoting public health services, and piloting public hospital reform [56, 57]. All the initiatives mentioned above might have contributed to the decrements. Moreover, the decline in inpatient care utilization was more obvious than the changes in self-care and outpatient care use, which could be associated with the NCMS reimbursement policies that focus on inpatient coverage [46]. Considering that primary care is more affordable and efficient in reducing health disparities [58], greater attention should be given to covering the costs of primary care through NCMS, such as the coverage of chronic disease management and treatment.

Our findings on trends in urban-rural disparities were consistent with Meng et al. [46]. However, earlier research conducted by Liu et al. and Gao et al. showed widening gaps in health care utilization between urban and rural from 1980s to 1993 and from 1993 to 1998, respectively [47] [59], which was opposite to our multivariate findings. There are several possible explanations for the discrepancy between their studies and our findings. First, Liu et al. and Gao et al. used secondary data sources without adjusting for confounders. Our results on trends of the raw urban-rural gaps had similar differences from the trends after controlling confounding variables. Second, Liu et al. and Gao et al. used living location to distinguish between urban and rural, while we defined urban and rural residents by hukou. During the past few decades, large-scale young people moved into cities leaving the elderly behind in the countryside, which might result in a more rapidly aging trend in rural areas compared to urban areas [12]. Most of these rural-to-urban migrants still held rural hukou and would not affect our results.

In addition, our findings suggested that the pattern of health seeking behaviours were different among different areas. For example, patients in central and eastern regions were less likely to use self-care and were more likely to use outpatient care, while patients in the north-eastern region were more intent on using self-care and were less likely to use professional health service. This reflected the uneven distribution of health resources among different parts of China [58]. Correspondingly, different policy interventions should be implemented to improve health care utilization accordingly. The government should improve the health infrastructure in undeveloped regions to encourage people to use health services when needed. For the eastern region, improving the primary care to disperse crowds of patients at big hospitals need further efforts.

### Limitations

The results from the present study should be interpreted with caution as there are some limitations. First, our data source did not include information on physician/provider practice style and the environmental factors related to health care utilization, which might be important in explaining rural–urban differences. Variation in provider side effects is an important area for further study. Second, health-related data including self-reported health status and some objective indicators were no longer available from CHNS since 2009, making it difficult to take the urban-rural health difference into consideration. Third, we did not assess quality and quantity of health care, or access to other sectors of the health care system, such as preventive services. Thus, an expanded study that examines disparities not only in health seeking behaviours but also in quantity and quality of health care usage needs to be conducted.

## Conclusion

Despite these limitations, this study is among the first to investigate the urban-rural disparities in health care seeking behaviours associated with hukou. Also, we used a longitudinal individual-level database including the most recent data, which explored some up-to-date findings on trends in urban-rural disparities. The present study showed that rural residents were still underutilizing health care when compared to their urban counterparts, and institutional differences between urban and rural in health care utilization were more notable than observed from statistical data. Fortunately, the Chinese government abolished the urban and rural hukou distinction in October 2014. Our findings attest to the wisdom of this abolishment. Although the urban-rural disparities have declined between 1993 and 2011, policy makers still need to be aware of the challenges due to aging problems and health expenditure increases. Therefore, a comprehensive approach to achieving and maintaining equity in health and heath care utilization may focus on: integrating the decentralized insurance system into one uniform system, providing more primary care services covered by health insurance, and equitable and reasonable allocation of health resources between urban and rural areas.

## Abbreviations

CHNS: China Health and Nutrition Survey
ISA: Individual saving accounts
MNL: multinomial logistic
NCMS: New cooperative medical system in rural
RRR: Relative risk ratios
UEBMI: Urban employee basic medical insurance
URBMI: Urban resident basic medical insurance
